# Will Sofosbuvir/Ledipasvir (Harvoni) Be Cost-Effective and Affordable for Chinese Patients Infected with Hepatitis C Virus? An Economic Analysis Using Real-World Data

**DOI:** 10.1371/journal.pone.0155934

**Published:** 2016-06-08

**Authors:** Guo-Feng Chen, Lai Wei, Jing Chen, Zhong-Ping Duan, Xiao-Guang Dou, Qing Xie, Wen-Hong Zhang, Lun-Gen Lu, Jian-Gao Fan, Jun Cheng, Gui-Qiang Wang, Hong Ren, Jiu-Ping Wang, Xing-Xiang Yang, Zhan-Sheng Jia, Qing-Chun Fu, Xiao-Jin Wang, Jia Shang, Yue-Xin Zhang, Ying Han, Ning Du, Qing Shao, Dong Ji, Fan Li, Bing Li, Jia-Liang Liu, Xiao-Xia Niu, Cheng Wang, Vanessa Wu, April Wong, Yu-Dong Wang, Jin-Lin Hou, Ji-Dong Jia, Hui Zhuang, George Lau

**Affiliations:** 1 Second Liver Cirrhosis Diagnosis and Treatment Center, 302 Hospital, Beijing, China; 2 Beijing Key Laboratory of Hepatitis C and Immunotherapy for Liver Diseases, Peking University People's Hospital, Peking University Hepatology Institute, Beijing, China; 3 Division of Gastroenterology & Hepatology, Humanity & Health Medical Centre, Hong Kong, Hong Kong SAR, China; 4 The Translational Hepatology Institue, Beijing You'an Hospital, Capital University of Medicine, Beijing, China; 5 Department of Infectious Diseases, Shengjing Hospital, China Medical University, Shenyang, China; 6 Department of Infectious diseases, Shanghai Ruijin Hospital, Jiaotong University, School of Medicine, Shanghai, China; 7 Key Laboratory of Medical Molecular Virology, Huashan Hospital, Fudan University, Shanghai, China Institute of Biomedical Sciences, Shanghai Medical College, Fudan University, Shanghai, China; 8 Department of Gastroenterology, Shanghai First People's Hospital, Shanghai Jiao Tong University School of Medicine, Shanghai, China; 9 Department of Gastroenterology, Xinhua Hospital, Shanghai Jiaotong University School of Medicine, Shanghai, China; 10 Liver Disease Center, Beijing Ditan Hospital, Capital University of Medicine, Beijing, China; 11 Department of Infectious Diseases, Center for Liver Diseases, Peking University First Hospital, Beijing, China; 12 Institute for Viral Hepatitis, Second Affiliated Hospital, Chongqing University of Medical Sciences, Chongqing, China; 13 Department of Infectious Disease, Xijing Hospital, Fourth Military Medical University, Xi'an, China; 14 Department of Infection, Sichuan Provincial People's Hospital, Chengdu, Sichuan, China; 15 Center of Diagnosis and Treatment for Infectious Diseases of Chinese PLA, Tangdu Hospital, Fourth Military Medical University, Xi’an, China; 16 Shanghai Liver Diseases Research Center, Nanjing Military Command, Shanghai, China; 17 People’s Hospital, Shanghai Jiaotong University, School of Medicine, Shanghai, China; 18 Department of Infectious Diseases, Henan Provincal People's Hospital, Zhengzhou University, Zhengzhou, China; 19 Department of Infectious Disease, Xinjiang Medical University First Affiliated Hospital, Urumqi, China; 20 Department of Digestive Diseases, Xijing Hospital, Fourth Military Medical University, Xi’an, China; 21 Liver Disease Center for Combined Traditional Chinese Medicine and Western Medicine, 302 Hospital, Beijing, China; 22 State Key Laboratory of Organ Failure Research, Guangdong Provincial Key Laboratory of Viral Hepatitis Research, Department of Infectious Diseases, Nanfang Hospital, Southern Medical University, Guangzhou, China; 23 Liver Disease Center, Beijing Friendship Hospital, Capital Medical University, Beijing, China; 24 Department of Microbiology and Center of Infectious Disease, School of Basic Medical Sciences, Peking University Health Science Center, Beijing, China; Chiba University, Graduate School of Medicine, JAPAN

## Abstract

**Background:**

Little is known on the cost-effectiveness of novel regimens for hepatitis C virus (HCV) compared with standard-of-care with pegylated interferon (pegIFN) and ribavirin (RBV) therapy in developing countries. We evaluated cost-effectiveness of sofosbuvir/ledipasvir for 12 weeks compared with a 48-week pegIFN-RBV regimen in Chinese patients with genotype 1b HCV infection by economic regions.

**Methods:**

A decision analytic Markov model was developed to estimate quality-adjusted-life-years, lifetime cost of HCV infection and incremental cost-effectiveness ratios (ICERs). SVR rates and direct medical costs were obtained from real-world data. Parameter uncertainty was assessed by one-way and probabilistic sensitivity analyses. Threshold analysis was conducted to estimate the price which can make the regimen cost-effective and affordable.

**Results:**

Sofosbuvir/ledipasvir was cost-effective in treatment-experienced patients with an ICER of US$21,612. It varied by economic regions. The probability of cost-effectiveness was 18% and 47% for treatment-naive and experienced patients, and it ranged from 15% in treatment-naïve patients in Central-China to 64% in treatment-experienced patients in Eastern-China. The price of 12-week sofosbuvir/ledipasvir treatment needs to be reduced by at least 81% to US$18,185 to make the regimen cost-effective in all patients at WTP of one time GDP per capita. The price has to be US$105 to make the regimen affordable in average patients in China.

**Conclusion:**

Sofosbuvir/ledipasvir regimen is not cost-effective in most Chinese patients with genotype 1b HCV infection. The results vary by economic regions. Drug price of sofosbuvir/ledipasvir needs to be substantially reduced when entering the market in China to ensure the widest accessibility.

## Introduction

In the last two years, new direct-acting antivirals (DAAs) were approved by the US Food and Drug Administration (FDA) including the first-in-class nucleotide polymerase inhibitor sofosbuvir (SOF), the NS5A inhibitor daclatasvir (DCV) as well as fixed-dose combinations of ledipasvir (NS5A inhibitor)/sofosbuvir. Their efficacy and safety, with and without the addition of pegylated interferon (pegIFN) and/or ribavirin (RBV), have been proven to be very high across patient groups in clinical trials[1–3]. However, the prices of these DAAs are prohibitively high and a lack of affordability has resulted in rationing of treatment in high-income countries such as the United States[4, 5]. Though recent studies in western developed countries have demonstrated that these new regimens appear to be cost-effective in most patient populations with hepatitis C virus (HCV) infections[6–17], there remains uncertainty about the cost-effectiveness of these new regimens in middle to upper-middle income Asian countries such as China.

China has largest number of HCV infection, with a minimum estimation of 10 million patients with chronic hepatitis C (CHC)[18]. Genotype 1b (GT 1b) is the most common genotype (56.8%)[19]. Current standard of care (SOC) is combination therapy with pegIFN and RBV for 48 weeks. This regimen is associated with relatively high sustained virological response (SVR) rates for Chinese adult patients (60–70%)[18]. However, interferon-based treatment is difficult to tolerate and has numerous contraindications. Roughly 40% of diagnosed CHC patients in China have been treated using interferon-based therapy.

DAAs are not current available in China. Clinical trials using DAAs have recently been initiated, and the market prices are not settled. China is a large and diverse country, with regions varying significantly in economic development (gross domestic product (GDP) per capita varies widely across the country) and socio-demographic profiles. The health financing system in China is quite decentralized, exacerbating rather than mitigating regional disparity. Though three public medical insurance programs (i.e. Urban Employee Basic Medical Insurance (UEBMI), Urban Resident Basic Medical Insurance (URBMI) and New Rural Cooperative Medical Scheme (NRCMS)) have covered 95% of the population by 2011[20], whether the treatment of CHC using pegIFN plus RBV is included in the public insurance programs has largely depends on the local financial status in each region[21]. All of these factors may lead to uncertainty around the cost-effectiveness of DAAs and risk uneven access and unaffordability to treatment if pricing does not take this variability into account. Understanding the cost-effectiveness of DAAs and its influencing factors at current market prices thus is of great importance in assisting the decisions on pricing, and expedite the approval of DAAs in the market.

Our study aimed to evaluate the cost-effectiveness of DAAs in Chinese patients with CHC GT1b, based on real-world practice in a Hong Kong-Beijing special hepatitis C clinic (302 Hospital, Beijing, China). We, taking fixed-dose combinations of sofosbuvir/ledipasvir (Harvoni®, Gilead) as an example, did the evaluation by four economic regions (Northeast, Central, East and West), assessed the budget impact and affordability of the regimen, and attempted to determine the price where the regimen is cost-effective and affordable from a government payer’s perspective.

## Materials and Methods

**Fig 1:**
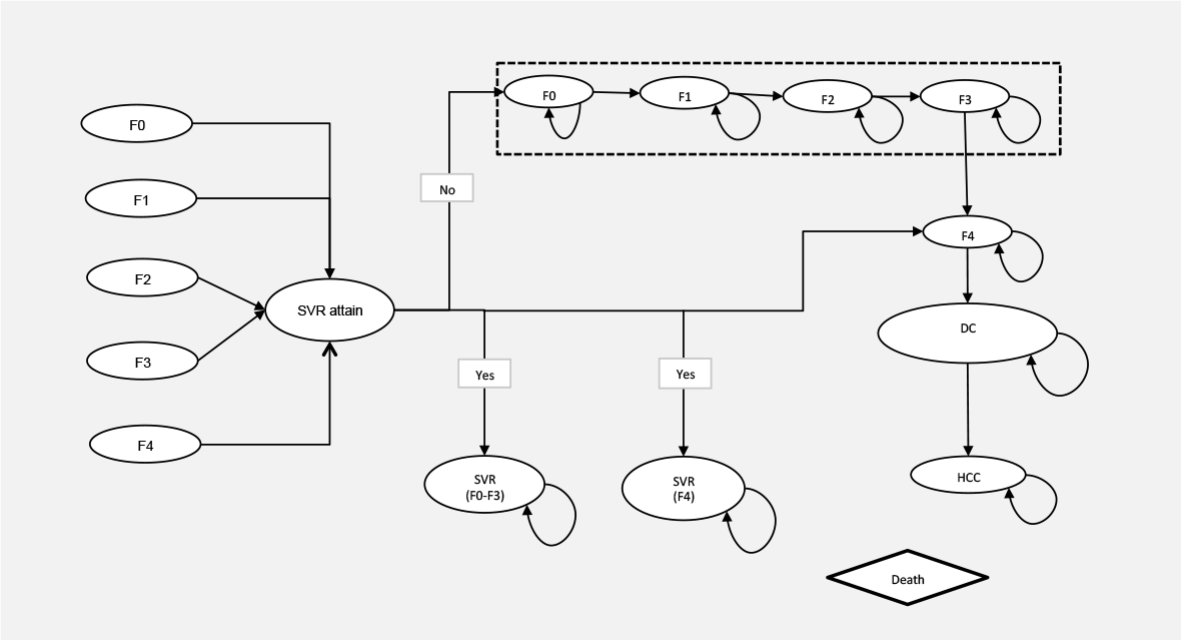
Simplified Markov Model. Subjects can progress through fibrosis stages F0-F4, DC based on natural progression rates. Fibrosis regression after SVR is possible for subject in stage F3 and F4. Further fibrosis progression to HCC, liver transplant after SVR is possible for subjects in stages F4 and DC at lower rates. DC, decompensated cirrhosis; F0-F4, Metavir fibrosis stages; HCC, Hepatocellular carcinoma.

A decision analytic Markov model based on the natural history of HCV infection in the published literature was developed to simulate the progression of a 50-year-old GT1b cohort by treatment history, sex and fibrosis stages (F0-F4 defined by METAVIR score[22]), using a lifetime horizon. Fig 1 represents the natural history of CHC infection. An annual discount rate of 3% was applied[23]. Our modeling approach and assumptions are described in detail in the supplementary materials.

### Treatment Effectiveness and Price

Since Chinese patients may respond differently compared with patients studied in global clinical trials of DAAs, effectiveness data was from a real-world practice in a Hong Kong-Beijing special hepatitis C clinic (302 Hospital, Beijing, China). The SVR rate was 100% for patients treated with Harvoni® regardless of treatment history and cirrhosis status. The 12-week drug price of Harvoni® was RMB600,000, reflected the actual price paid by patients. The 48-week drug price of pegIFN and RBV was RMB46,447. Both the prices were converted to 2014 U.S. dollars (Table 1). All patients were treated once within a one-year time frame assuming no discontinuation and no retreatment would occur. The detailed information of patients in this clinic is described in supplementary materials.

**Table 1:** Model transition probabilities, cost inputs and utilities.

Input parameter	Mean (Range)	Distribution (a, b)	Reference
**SVR rates**	** **	** **	** **
**Treatment Naive**			
PegIFN+RBV without cirrohsis	0.75		Real data from SOPC
PegIFN+RBV cirrohsis	0.38		ibid
SOF-LDV without cirrhosis	1 (0.9–1)	Uniform (0.9, 1)	ibid
SOF-LDV with cirrhosis	1 (0.9–1)	Uniform (0.9, 1)	ibid
**Treatment Experienced**			
PegIFN+RBV without cirrohsis	0.23		Real data from SOPC
PegIFN+RBV cirrohsis	0.11		ibid
SOF-LDV without cirrhosis	1 (0.9–1)	Uniform (0.9, 1)	ibid
SOF-LDV with cirrhosis	1 (0.9–1)	Uniform (0.9, 1)	ibid
**Distribution of METAVIR fibrosis stages at baseline**			
F0	0.17 (0.13–0.21)	Uniform (0.13,0.21)	Thein, 2008 (27)
F1	0.35 (0.26–0.44)	Uniform (0.26,0.44)	ibid
F2	0.22 (0.17–0.27)	Uniform (0.17,0.27)	ibid
F3	0.14 (0.10–0.17)	Uniform (0.19,0.17)	ibid
F4	0.12 (0.09–0.15)	Uniform (0.09,0.15)	ibid
**Annual Transition Probabilities**			
***Stage progression probabilities***			
F0-F1*	0.117 (0.104–0.13)	β (275, 2075)	Thein, 2008 (27); Chhatwal, 2015 (10)
F1-F2*	0.085 (0.075–0.096)	β (210, 2261)	ibid
F2-F3*	0.12 (0.109–0.133)	β (288, 2112)	ibid
F3-F4*	0.116 (0.104–0.129)	β (271, 2062)	ibid
F4-DC	0.038 (0.031–0.051)	β (59, 1447)	Najafzadeh, 2015 (13)
F4-HCC	0.021 (0.017–0.028)	β (40, 1887)	ibid
DC-HCC	0.021 (0.017–0.028)	β (40, 1887)	ibid
DC—death (1st year)	0.26 (0.12–0.33)	Uniform (0.12,0.33)	Saab, 2014 (8)
DC—death (subsequent year)	0.26 (0.12–0.33)	Uniform (0.12,0.34)	ibid
HCC—death	0.349 (0.288–0.4)	Uniform (0.288, 0.40)	Hagan, 2014 (6)
RR of post SVR F4-DC	0.13 (0.06–0.27)	Lognormal (-2.04, 0.38)	Singal 2010 (28)
RR of post SVR F4/DC-HCC	0.32 (0.23–0.44)	Lognormal (-1.14, 0.16)	ibid
***Stage regression probabilities***			
F3-F2 post-SVR	0.393 (0.221–0.56)	Uniform (0.22, 0.56)	Hagan, 2014 (6)
F4-F2 post-SVR	0.077 (0.058–0.095)	Uniform (0.06, 0.09)	ibid
F4-F3 post-SVR	0.288 (0.173–0.387)	Uniform (0.17, 0.39)	ibid
**Cost inputs (US$)**		
***Treatment***			
PegIFN+RBV (48 weeks)	7488 (5616–7488)	Gamma (1, 7488)	Real life data from SOPC
SOF-LDV (12 weeks)	96729 (72547–96729)	Gamma (1, 96729)	ibid
***Annual direct medical costs of each health state***		Real life data from main hospitals in eight cities in Mainland China	
***Northeastern***			
F0-F3	131 (98–164)	Gamma (1, 131)	
F4	131 (98–164)	Gamma (1, 131)	
DC	2435 (1826–3043)	Gamma (1, 2435)	
HCC	14608 (10956–18261)	Gamma (1, 14608)	
***Central***			
F0-F3	836 (627–1045)	Gamma (1, 836)	
F4	3023 (2267–3779)	Gamma (1, 3023)	
DC	2597 (1948–3246)	Gamma (1, 2597)	
HCC	5113 (3835–6391)	Gamma (1, 5113)	
Eastern*			
F0-F3	1151 (801–1501)	Gamma (10, 111)	
F4	3223 (-49-6495)	Gamma (1, 3459)	
DC	7698 (4165–11230)	Gamma (5, 1688)	
HCC	14920 (10724–19115)	Gamma (12, 1229)	
***Western***			
F0-F3*	883 (547–1220)	Gamma (13, 131)	
F4	2224 (1668–2780)	Gamma (1, 131)	
DC	4869 (3652–6087)	Gamma (1, 2435)	
HCC	6493 (4869–8116)	Gamma (1, 14608)	
All China*			
F0-F3	917 (620–1214)	Gamma (5, 200)	
F4	2610 (925–4295)	Gamma (1, 2266)	
DC	5813 (3532–8094)	Gamma (3, 1864)	
HCC	12270 (8824–15717)	Gamma (6, 2016)	
RR of cost post SVR*	0.709 (0.592–0.855)	Lognormal (-0.34, 0.09)	Manos, 2013 (29)
**Utilities**			
F0-F3	0.81 (0.61–1)	Uniform (0.61, 1)	Thein, 2005 (30)
F4	0.76 (0.57–0.95)	Uniform (0.57, 0.95)	ibid
DC	0.69 (0.52–0.86)	Uniform (0.52, 0.86)	ibid
HCC	0.67 (0.5–0.84)	Uniform (0.5, 0.84)	ibid
Post SVR	0.87 (0.65–1)	Uniform (0.65, 1)	ibid
DDA treatment decrement*	0.95 (0.9–1)	β (86, 10)	Chhatwal, 2015 (10)
IFN treatment decrement*	0.9 (0.84–0.96)	β (108, 6)	ibid

* Range was reported as 95%CI; Without indication, range was reported as ±25%.

SOF = sofosbuvir, pegIFN = pegylated interferon, RBV = ribavirin, DC = decompensated cirrhosis, HCC = Hepatocellular carcinoma.

The retrospective study was approved by Hong Kong Clinical Research Ethics Committee (HKCREC), Hong Kong. The patients record and information was anonymized and de-identified prior to the analysis.

### Mortality Rates

Age-specific background mortality rates in China were used in each model[24, 25]. We used a mortality rate ratio of 2.37 from US population[26] in our model for patients in stage F0-F4 as we lack this data in Chinese populations and we tested it in sensitivity analyses. Patients with decompensated cirrhosis (DC) and hepatocellular carcinoma (HCC) were assigned specific death rates[6, 8, 13] (Table 1). Since liver transplant was not a usual practice in China (1,897 cases in 2011[18]), we did not include it in our model.

### Transition Probabilities

Baseline fibrosis before treatment and annual progression probabilities were obtained from a meta-analysis[27]. To account for liver regeneration after SVR, post-SVR fibrosis regression was modeled[6]. Reduced post-SVR progression rates to DC and HCC from stage F4, and to HCC from DC were modeled to reflect the decreased progression risks as evidence showed[28] (Table 1).

### Direct Medical Costs

In China, a large share of outpatient visits is to secondary and tertiary hospital outpatient departments which are government owned and managed. Bureau of Commodity Prices determines the prices for medical assessments and treatments in these hospitals in each province. For the same medical procedure, the prices within a city are relatively uniform across all hospitals. We obtained annual direct medical costs associated with disease states F0-F4, DC, and HCC from major hospitals in each economic region: Northeast (Shenyang), Central (Zhengzhou), East (Beijing, Shanghai and Guangzhou), and West (Chongqing, Xi’an, Chengdu, and Urumqi). Annual medical costs included costs for liver-related and other laboratory tests (i.e. plasma HCV RNA level, alpha fetoprotein, IL28B (rs12979860), IFNL4 (rs368234815), genotyping, complete blood count, comprehensive metabolic panel, thyroid-stimulating hormone, PT-INR), procedures (liver biopsy, liver ultrasound, gastroscopy), medications, and hospitalizations. Costs that were not available from a particular city were replaced with the mean cost for that region. It was found that healthcare costs were significantly lower for patient with SVR than for those without SVR[29]. We assumed that direct medical costs were reduced in patients in stage F3-F4 who achieved SVR. No direct medical costs were born by patients in stage F0-F2 who achieved SVR. Costs due to drug intake, medical visits, and hospital admissions from treatment-associated adverse events were not included to avoid double counting since the impact of treatment toxicity was considered in health-related quality of life measured as utility values. All costs were converted to 2014 U.S. dollars. Mean cost of the four regions was used to estimate the cost-effectiveness for all China (Table 1).

### Quality-of-Life Weights (Utility)

Because utility values for Chinese patients were not available, we used the values for each disease state from a systematic review including 19 studies with direct HRQOL assessment using the Short Form-36 in HCV patients[30]. Utility decrements during SOC and sofosbuvir/ledipasvir were assigned to take into account the reduced HRQOL due to treatment-related adverse events (Table 1).

### Cost-Effectiveness Analysis

The incremental cost-effectiveness ratio (ICER) was calculated by dividing the difference in lifetime costs between sofosbuvir/ledipasvir and SOC by the difference in quality-adjusted life-years (QALYs), separate for treatment-naïve and -experienced patients with and without cirrhosis. We do not have a determined willingness-to-pay (WTP) threshold as that in the U.S. (US$50,000) or U.K. (US$45,953). According to WHO guidelines[31], three times gross domestic product (GDP) per capita in each region and in China was considered as the maximum threshold limit for cost-effectiveness.

We calculated the life expectancy from different disease states (non-cirrhosis and cirrhosis) and the percentage of patients who progressed to cirrhosis, and compared them with published data to validate the model[9, 32]. Details of model validation are described in supplementary materials.

### Sensitivity Analysis

One-way sensitivity analysis was conducted to assess the impact of changes of individual model parameter and assumptions on the model results. Whenever available, the 95% confidence interval (95%CI) reported were used (Table 1). Otherwise, the ranges of the estimates reported were used. If none was available, we varied the parameter by ±25%. Probabilistic sensitivity analysis using second order Monte Carlo simulation was performed to estimate the variance and distribution of the cost effectiveness estimates. The model was simulated on 1,000 iterations by randomly sampling values from the distributions assigned to each parameter. The simulation results are presented by means of cost-effectiveness acceptability curves. The model was built using Excel 2011.

### Budget Impact and affordability Analysis

We estimated the budget required to treat all eligible patients in China which are a minimum of 10 million patients[18]. By using the mean costs generated from our model, we estimated the resources needed to treat these 10 million patients with sofosbuvir/ledipasvir.

We evaluated the affordability of sofosbuvir/ledipasvir using the average daily wage of urban employees. As suggested by WHO, treatments costing one day’s wage or less for a full course of treatment for an acute condition or a 30-day supply of medicine for chronic diseases are generally considered affordable[33].

### Price threshold analysis

In order to assess the impact of price of sofosbuvir/ledipasvir on the cost-effectiveness, we performed a threshold analysis. Keeping all the other parameters constant, we estimated the maximum price where the regimen would be cost-effective compared to SOC. The price where the average patients can afford was also estimated.

## Results

### Base-Case Analysis

Depending on the treatment history, cirrhosis status, and economic regions, the ICERs ranged from US$8,950 to US$89,470 per QALY gain when comparing sofosbuvir/ledipasvir to SOC (Table 2). By the WHO guideline of 3 times GDP per capita, sofosbuvir/ledipasvir was cost-effective in cirrhotic patients in Northeastern-, Central- and Western-China, and cost-effective in treatment-experienced patients in Eastern-China.

**Table 2:** ICERs of sofosbuvir/ledipasvir compared to pegIFN plus RBV, by four economic regions in China.

	All China	Northeastern	Central	Eastern	Western
**GDP per capita**	7590	8490	6065	10928	6095
**Treatment Naïve**					
All patients	58958	62296	59489	57794	59692
No cirrhosis	85588	89471	86061	84319	86219
Cirrhosis	15975	18434	16598	14980	16876
**Treatment Experienced**					
All patients	21612	25256	22130	20377	22319
No cirrhosis	25067	29061	25554	23761	25716
Cirrhosis	9947	12413	10572	8950	10851

### Sensitivity Analysis

Using one-way sensitivity analysis, we identified 10 parameters that had the largest impact on ICERs comparing sofosbuvir/ledipasvir to SOC for each region (S1 Fig). The ICERs were most sensitive to post-SVR utility score, price of sofosbuvir/ledipasvir, discount rate on costs, and utility scores associated with fibrosis stages F1-F4.

**Fig 2:**
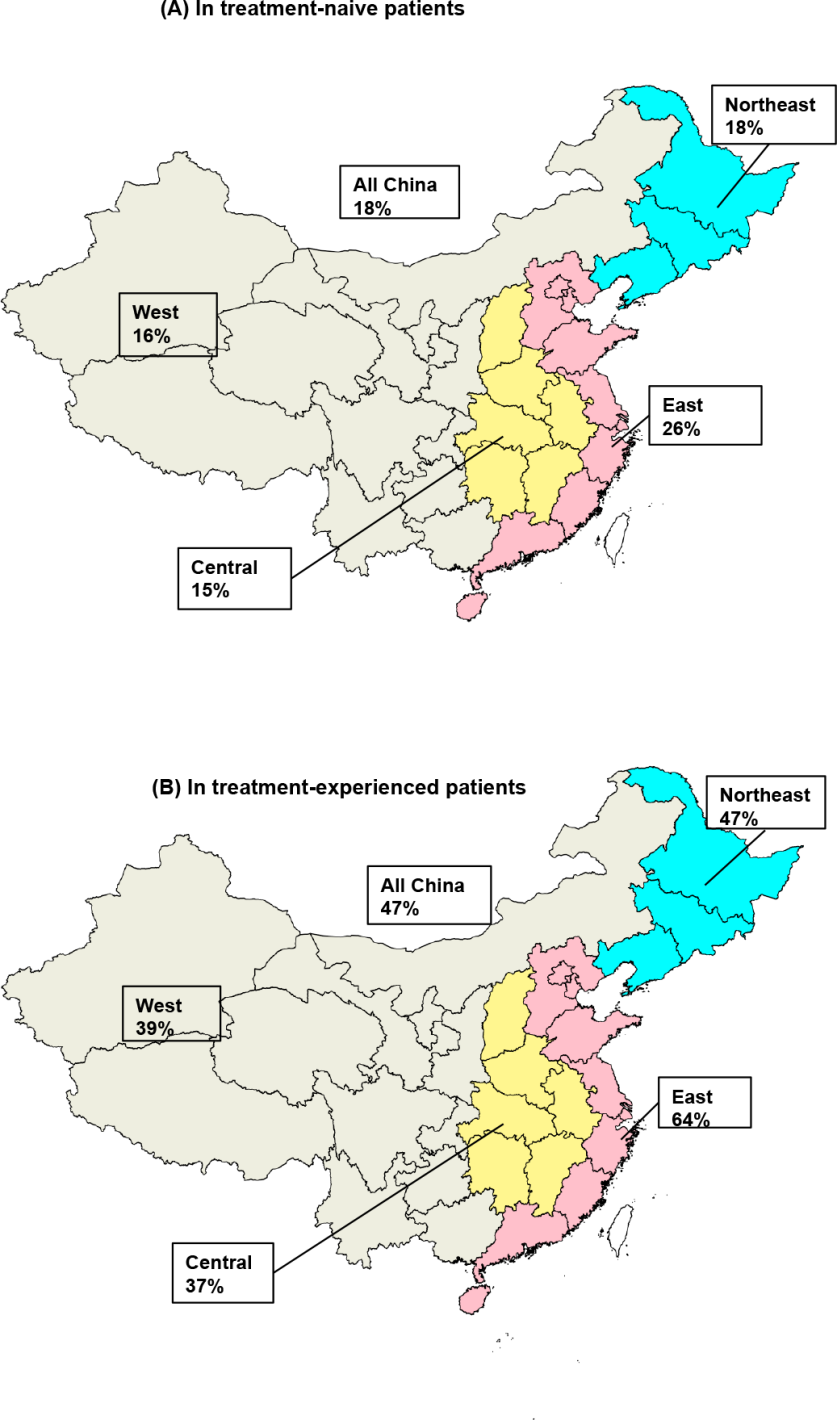
**Probability of Cost-effectiveness in A: Treatment-Naïve and B: Treatment-Experienced patients, by economic regions in China.** The probability was at the willingness-to-pay of 3 GDP per capita in each region.

Probabilistic sensitivity analysis showed that the probability that sofosbuvir/ledipasvir was cost-effective at a US$22,770 WTP was 18% and 47% for treatment-naïve and -experienced patients respectively in China (Fig 2). The probability varied by regions. In treatment-experienced patients, the probability of cost-effectiveness for sofosbuvir/ledipasvir was less than 50% except for that in Eastern-China (64%).

### Budget impact analysis and affordability

We estimated that 5.3 million GT1b CHC patients would be eligible for treatment with a 95% health insurance coverage[20]. Among 5.3 million eligible patients, 1.5 million are with cirrhosis. A total of US$522 billion would be needed to cover the drug cost for all eligible patients, and US$261 billion would need to be paid by government one-off if the public health insurance programs cover 50% of the drug cost. When only patients with cirrhosis were covered, a total of US$73 billion would need to be paid by government. Using the mean costs estimated from our model, compare to SOC, sofosbuvir/ledipasvir would cost government an additional US$202 billion including drug costs and direct medical costs if the public health insurance programs cover 50% of these costs.

The average daily wage for urban employees in China in 2014 was US$35. Averagely, an employee needs to work 2,776 days (96972/35) to receive a 12-week treatment of sofosbuvir/ledipasvir (or 923 days to purchase one bottle of sofosbuvir/ledipasvir). It is far above the affordable benchmark indicated by WHO.

### Price threshold analysis

The price of 12-week sofosbuvir/ledipasvir treatment needs to be reduced by at least 69% to US$29,793 make the regimen cost-effective in all patients at WTP of three times GDP per capita (Table 3). The price reduction was 81% to US$18,185 if WTP was one time GDP per capita (Table 3). If complying with the WHO benchmark, the price of sofosbuvir/ledipasvir per bottle cannot exceed US$35 (US$105 for a 12-week course).

**Table 3:** Prices that make sofosbuvir/ledipasvir cost-effective, by four economic regions in China.

	**Threshold limit at three GDP per capita**				
**Treatmnet Naïve**	**All China**	**Northeastern**	**Central**	**Eastern**	**Western**
**All patients**	45946 (52)	44979 (53)	38788 (60)	61616 (36)	38595 (60)
**No cirrhosis**	34822 (64)	33662 (65)	29889 (69)	45946 (52)	**29793 (69)**
**Cirrhosis**	—	—	—	—	—
**Treatment Experienced**					
**All patients**	—	—	83477 (14)	—	83090 (14)
**No cirrhosis**	89861 (7)	86089 (11)	74965 (22)	—	74772 (23)
**Cirrhosis**	—	—	—	—	71579 (26)
	**Threshold limit at one GDP per capita**				
**Treatmnet Naïve**	**All China**	**Northeastern**	**Central**	**Eastern**	**Western**
**All patients**	24596 (75)	21184 (78)	26020 (73)	30953 (68)	21474 (78)
**No cirrhosis**	19926 (80)	**18185 (81)**	26020 (73)	24472 (75)	21184 (78)
**Cirrhosis**	59198 (39)	52234 (46)	49525 (49)	78544 (19)	48461 (50)
**Treatment Experienced**					
**All patients**	49525 (49)	40336 (58)	42657 (56)	64905 (33)	42174 (56)
**No cirrhosis**	45172 (54)	36080 (63)	39272 (60)	58811 (39)	38885 (60)
**Cirrhosis**	81543 (16)	71579 (26)	67807 (30)	—	66259 (31)

Results were presented as price (reduction %);—means it was cost-effective within the threshold limit

## Discussion

Our cost-effectiveness analysis of sofosbuvir/ledipasvir in Chinese CHC GT1b patients by regions shows that the regimen might not be cost-effective in all patient groups in different economic regions relative to their GDP per capita. Price is the major factor influencing the cost-effectiveness. At current price, neither government nor individual can afford the drug. The price for a 12-week course of treatment needs to be reduced by at least 81% to US$18,185 from current market price to make the regimen cost-effective.

High prices is the main barrier to accessibility to affordable DAAs, especially in upper-middle-income countries like China[34]. High-income countries are able to negotiate with pharmaceutical companies with lower prices and their citizens are either fully covered by national reimbursement (e.g. Australia) or need only to pay small amount out-of-pocket fee (US$300 in Japan and US$2000 in Korea) to get treated. Most lower-middle-income and low-income countries are included in the voluntary license agreements by pharmaceutical companies like Gilead and Bristol-Myers Squibb, with access to low-price licensed generic drugs. Upper-middle-income countries like China where most of the HCV burden lies are not only not included in the voluntary license agreements but also unable to fund the purchase of DAAs, which restrict their accessibility to affordable DAAs. As our analysis showed that huge financial burden (US$522 billion) would be posed to government if DAAs entered the market at current market price. Even if only patients with cirrhosis were treated, in order to maximize clinical benefit, treatment would need to occur over a short time frame and would still dramatically affect the government’s financial resources.

As our analysis showed that substantial reduction (at least 81% to US$18,185) from current market price of sofosbuvir/ledipasvir (US$96,729) is required to make the regimen cost-effective in all patients. A further reduction is needed to ensure the access to affordable treatment for each individual (down to US$105 as estimated). Price needs to be carefully settled when DAAs enter China market. Because of the restriction on accessibility to DAAs currently in China, patients seek for low-priced products by illegal means. They can either purchase the DAAs sold online or join the tourist groups to the neighboring countries like India, where generic products are available, to purchase the DAAs in person. It undermines the license agreement between generic manufactures and the pharmaceutical companies, and has potential risk of jeopardizing patients’ health if the drug is counterfeited as WHO recently alerted[35].

In order to get access to affordable DAAs, the government needs to negotiate with pharmaceutical companies with much lower prices to afford the purchase and include them in the National Essential Medicines List as WHO did[36]. In order to do so, government needs to conduct national screening on HCV to understand the true burden of the disease, explore the widest public market and use the economics of scale to achieve the lowest prices. Also, the government could make use of the flexibilities under the World Trade Organization’s agreement on Trade-Related Aspects of Intellectual Property Rights (TRIPS) to issue compulsory licenses to enable local production of generic version. It is of important relevance to countries that have capacity to produce raw materials and finished products of DAAs like China who in fact is the largest provider of raw materials to generic manufactures in India and Pakistan. Further, shorten the treatment duration in clinical settings. A couple of phase 2 studies have demonstrated high SVR12 rates by 6- to 8-week DAAs treatment[37, 38]. One recent phase 2 study has further shortened the treatment to 3-weeks and resulted in a 100% SVR12[39]. Shorter therapy will reduce the price of DAAs by up to two-thirds without affecting SVR rates, allowing more people to be treated promptly.

The cost-effectiveness of the new DAAs treatment relies on the society’s willingness to pay for improvements in health. As suggested by WHO, we adopted a threshold of three times GDP per capita as the decision rule in our analysis[31], although a threshold of one times per capita GDP is considered highly cost effective and is more in line with what is being used in the U.S. and Western Europe for HCV treatment[40]. If one GDP per capita was used, the price of sofosbuvir/ledipasvir needs to be further reduced by at least 81% to make the regimen cost-effective. Also, cost-effectiveness cannot reflect the affordability. If WHO benchmark of price was used, the price of sofosbuvir/ledipasvir was far from affordable which is the reality in China. This criterion of 3 times GDP per capita needs to be reconsidered in the evaluation of HCV as the price of the drug is exceptionally high.

Our study has several limitations. We lacked Chinese-specific data for many parameters and had to rely on estimates from the published literature. Small sample size for the primary efficacy measurement could have contributed to parameter uncertainty with respect to the expected SVR rate. But our analysis showed that even with a 100% SVR rate, sofosbuvir/ledipasvir had low probabilities to be cost-effective in most patients. We restricted our analysis to direct medical costs and did not include indirect costs (e.g. productivity loss) and non-medical costs (e.g. costs and time for patients to seek medical care, costs to hire care givers). But this may lead to estimates that are more conservative. We did not model the future possibility of retreatment with the new regimens (either due to treatment failure or reinfection) nor the potential benefit of preventing incident HCV infectious through reductions in the number of infected patients from the population. In addition, the natural history after treatment with these new regimens is uncertain but we have comprehensively tested the natural history parameters in our sensitivity analyses.

Our analysis demonstrated that sofosbuvir/ledipasvir might not be cost-effective in all Chinese GT1b patients infected with HCV at current price. At current price, it would pose a huge burden to government’s healthcare finance. Drug price of sofosbuvir/ledipasvir needs to be substantially reduced when entering the market in China. Hopefully current exercise will set a benchmark for upper-middle-income Asian countries to settle the price of DAAs and to ensure the widest access to DAAs.

## Supporting Information

S1 Fig Tornado diagram of one-way sensitivity by previous treatment history in A: All China, B: Northeastern-China, C: Central-China, D: Eastern-China and E: Western-China.
(DOCX)

S1 File More details on the model. Model validation: life expectancies for chronic liver disease stage and general population (Table A). Validation of the natural history of our model (Table B).
(DOCX)

## Abbreviations

DAAs: Direct-acting antivirals
FDA: Food and Drug Administration
SOF: sofosbuvir
DCV: daclatasvir
pegIFN: pegylated interferon
RBV: ribavirin
HCV: hepatitis C virus
CHC: chronic hepatitis C
GT 1b: Genotype 1b
SOC: standard of care
SVR: sustained virological response
DC: decompensated cirrhosis
HCC: hepatocellular carcinoma
ICER: Incremental cost-effectiveness ratio
WTP: willingness-to-pay
GDP: gross domestic productTRIPS: Trade-Related Aspects of Intellectual Property Rights
