# Supplementary material for: Will Sofosbuvir/Ledipasvir (Harvoni) Be Cost-Effective and Affordable for Chinese Patients Infected with Hepatitis C Virus? An Economic Analysis Using Real-World Data
Source: PLoS One. 2016 Jun 8;11(6):e0155934. doi: 10.1371/journal.pone.0155934 (PMC4898683; doi:10.1371/journal.pone.0155934)
Supplement: S1 Fig — Tornado diagram of one-way sensitivity by previous treatment history in A: All China, B: Northeastern-China, C: Central-China, D: Eastern-China and E: Western-China. (DOCX) [file pone.0155934.s001.docx]

**A All China**


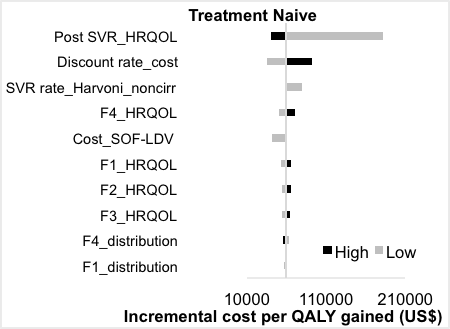

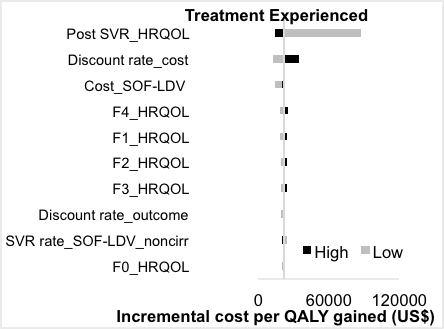


**B. Northeastern-China**


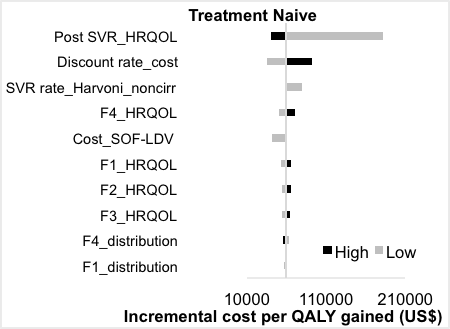

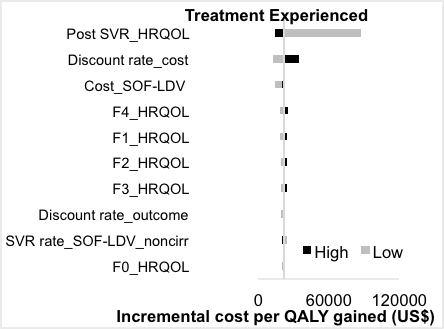


**C. Central-China**


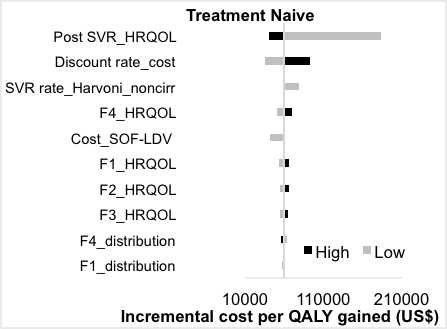

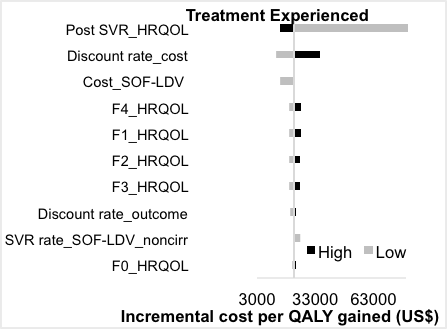


**D. Eastern-China**


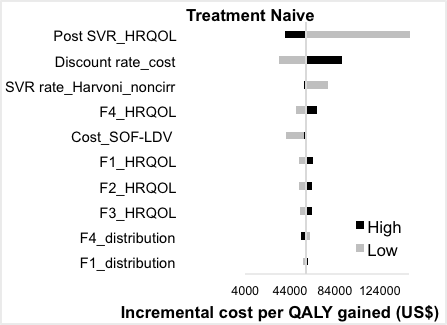

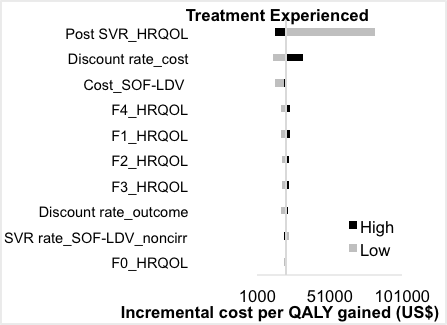


**E. Western-China**


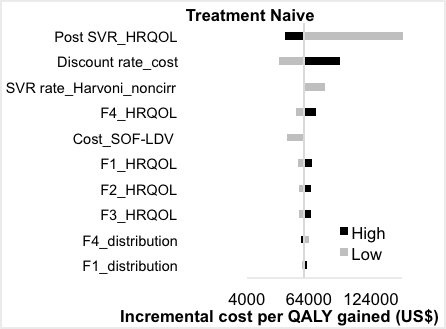

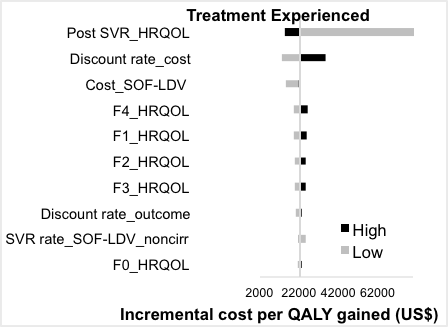


**S1 Fig. Tornado diagram of one-way sensitivity by previous treatment history in A: All China, B: Northeastern-China, C: Central-China, D: Eastern-China and E: Western-China.**

Abbreviations: Post SVR_HRQOL, quality of life after achieving sustained virologic response (SVR); Discount rate_cost: discount rate on the cost; Discount rate_outcome: discount rate on the outcome; SVR rate_SOF-LDV_cirr: SVR rate of the treatments in patients with cirrhosis; SVR rate_SOF-LDV_noncirr: SVR rate of the treatments in patients without cirrhosis; Cost_SOF-LDV: cost of treatment with SOF-LDV; RR of cost after SVR: relative risk of direct medical cost after successfully treated; F1_HRQOL, F2_HRQOL, F3_HRQOL, F4_HRQOL: utility score associated with F1-F4; F4_distribution: baseline distribution of F4;DC_LT transition: probability of patients in DC to have liver transplant; DC-death (1st year)_transition: transition rate of DC to death in the first year.
