# Supplementary material for: Will Sofosbuvir/Ledipasvir (Harvoni) Be Cost-Effective and Affordable for Chinese Patients Infected with Hepatitis C Virus? An Economic Analysis Using Real-World Data
Source: PLoS One. 2016 Jun 8;11(6):e0155934. doi: 10.1371/journal.pone.0155934 (PMC4898683; doi:10.1371/journal.pone.0155934)
Supplement: S1 File — Model validation: life expectancies for chronic liver disease stage and general population (Table A). Validation of the natural history of our model (Table B). (DOCX) [file pone.0155934.s002.docx]

**Supporting Information**

(References cited in appendix are referred to manuscript references lists)

**More details on the model**

**Model structure and Assumptions**

We developed a Markov model using Excel 2011 to simulate the natural history and progression of the disease among treatment-naïve and -experienced patients infected with chronic HCV genotype 1b and compare health and economic outcomes of different treatment regimens. We created a hypothetical cohort of 10 000 patients with baseline characteristics (sex, fibrosis stage based on METAVIR score, and treatment history) to mimic the actual distribution of patients with HCV in China. Patients started the model at age 50 with different levels of fibrosis (F0-F4). Each subsequent year, they could progress to a higher METAVIR stage (F0-F4) or, develop decompensated cirrhosis and hepatocellular carcinoma. After achieving sustained virologic response (SVR), it is assumed that patients in stage F0-F3 would not progress to an advanced stage. The progression rates to decompensated cirrhosis (DC) or hepatocellular carcinoma (HCC) among patients in stage F4 would be reduced. The progression rate to HCC among patients in stage DC would be reduced.

At the end of each model year, costs and quality-adjusted life years (QALYs) associated with disease state were accumulated across patients during that year. Death was possible from any state. Patients alive at the end of a given state would continue to progress to next state as determined by their previous disease state or treatment outcome. Those who are alive continued to accumulate costs and QALYs each cycle year till death.

By creating identical clones of the hypothetical cohort and assigning them to different treatment regimens, we compared health and economic outcomes with standard of care. Both QALYs and costs were discounted at a rate of 3% per year and sensitivity analysis was conducted with 0% and 6% discount rates. The cost-effectiveness analysis was conducted from a perspective of government payer. Costs and QALYs of sofosbuvir/ledipasvir compared with standard of care were presented in an incremental cost-effectiveness ratio.

The SVR rates were derived from our real-world practice in our Hong Kong-Beijing special hepatitis C clinic (302 Hospital, Beijing, China). By the end of April 2015, the SOPC treated 138 patients with genotype 1b HCV infection in fibrosis stage F0-F4 from China Mainland using SOF-based regimens for 12 weeks, in which 69 patients were treated with SOF-LDV (Harvoni®).

Treatment-Naïve patients

Ten patients were treated with SOF-LDV (Harvoni®) for 12 weeks and the SVR 12 rate was 100% in both non-cirrhotic (8/8) and cirrhotic (2/2) patients.

Treatment-experienced patients

Meanwhile, 59 patients were treated with Harvoni® for 12 weeks and the SVR 12 rate was 100% in both non-cirrhotic (29/29) and cirrhotic (30/30) patients.

In our real-world practice, there was no relapser 12 weeks or more after completion of the treatment. We assumed that there was no relapser. Also, for those who did not achieve SVR 12 week post-treatment, there was no retreatment for them. Treatment costs were based on the charges for patients who were treated in the special clinic. To test the uncertainty around the SVR 12 rates, one-way sensitivity analysis was conducted.

We modeled excessive mortality rates among patients with HCV in fibrosis stage F0-F4 based on whether they achieved SVR or not. Those who achieved SVR would be assumed to have the same mortality rate as that in general population but those who did not achieve SVR would have an excessive mortality rate than general population. We derived from published models the probabilities of liver-related mortality in patients with DC and HCC.

Whenever the transition probabilities were presented as the rates, we used the formula: *p=1-exp(-rt)* to convert the rates to annual transition probabilities, where p denotes the probability, r denotes the rate and t denotes the time.

Monte Carlo simulation was used to account for second-order uncertainty in our probabilistic sensitivity analysis. We sampled 1,000 times of the input parameters from their probability distribution. Beta, lognormal and gamma distribution were used for parameters of transition probabilities, relative risks and costs. If no sufficient data can be used for above distributions, uniform distribution was used based on ranges instead.

**Model validation**

The face validation in our model was done by setting certain parameters, e.g. SVR rate, cost of treatments at null or extreme values to check whether the model generated the predictable outcomes. To confirm the external validity, we made comparisons between outcomes from the model and the empiric data from published literature.

We first calculated the life expectancies from different states under standard of care (Table A). Averagely, life expectancies of patients with non-cirrhosis (F0-F3) and cirrhosis (F4) were shortened by 5 and 11 years than general population. The differences are comparable to those of other models (13). The survival of patients with SVR is similar to that of the general of population.

We then compared the 10-year cumulative incidence rates for DC and HCC predicted from our model with the published literatures (Table B). We compared the cumulative incidence for METAVIR score F4 to DC and HCC for those who failed to achieve SVR and those who achieve SVR separately. Compared with van der Meer et al (34), the predicted 10-year cumulative incidence rates for DC were within the reported confidence intervals of their study. The predicted 10-year cumulative incidence rates for HCC were slightly lower than the reported confidence intervals. Part of the differences could be potentially attributed to differences in baseline patients’ histologic status and the use of a different scoring system (Ishak score vs. METAVIR) in van der Meer study and our model.

**Table A. Model validation: life expectancies for chronic liver disease stage and general population**

|  | **General population** | | **Non-cirrhosis** | | **Cirrhosis** | |
| --- | --- | --- | --- | --- | --- | --- |
| **Age 50** | **M** | **F** | **M** | **F** | **M** | **F** |
| **Treatment-naïve** | 26.9 | 29.4 | 24.4 | 25.9 | 17.8 | 18.9 |
| **Treatment-experienced** | 26.9 | 29.4 | 20.7 | 22.5 | 14.7 | 15.7 |

**Table B. Validation of the natural history of our model**

| **Treatment response** | **Disease stage** | **10-year cumulative incidence** | |
| --- | --- | --- | --- |
|  |  | **van der Meer et al** | **Model** |
| **Patients who failed to achieve SVR** | DC | 29.9% (95%CI: 24.3-35.5%) | 28.4% |
|  | HCC | 21.8% (95%CI: 16.6-27.0%) | 15.2% |
| **Patients who achieved SVR** | DC | 2.1% (95%CI: 0-4.5%) | 1.3% |
|  | HCC | 5.1% (95%CI: 1.3-8.9%) | 1.1% |
